# Internet-Based Information Behavior After Pregnancy Loss: Interview Study

**DOI:** 10.2196/32640

**Published:** 2022-03-02

**Authors:** Nazanin Andalibi, Kristen Bowen

**Affiliations:** 1 School of Information University of Michigan Ann Arbor, MI United States; 2 University of North Carolina at Chapel Hill Chapel Hill, NC United States

**Keywords:** pregnancy loss, miscarriage, reproductive health, stigma, information behavior

## Abstract

**Background:**

Information behavior describes all human behaviors in relation to information. Individuals experiencing disruption or stigma often use internet-based tools and spaces to meet their associated information needs. One such context is pregnancy loss, which, although impactful and common, has been absent from much of feminist and reproductive health and information behavior scholarship. By understanding information behavior after pregnancy loss and accounting for it in designing internet-based information spaces, we can take a meaningful step toward countering the stigma and silence that many who experience such loss endure, facilitate coping, and make space for diverse pregnancy narratives in our society.

**Objective:**

This study’s objective is to provide a characterization of internet-based information behavior after pregnancy loss.

**Methods:**

We examined internet-based information behavior after pregnancy loss through 9 in-depth interviews with individuals residing in the United States. We analyzed the data by using open and axial coding.

**Results:**

We identified the following three themes in relation to participants’ information behavior in internet-based spaces: needed information types, information-related concerns, and information outcomes. We drew from information behavior frameworks to interpret the processes and concerns described by participants as they moved from recognizing information needs to searching for information and to using information and experiencing outcomes. Specifically, we aligned these themes with information use concepts from the information behavior literature—information search, knowledge construction, information production, information application, and information effects. Participants’ main concerns centered on being able to easily find information (ie, searchability), particularly on topics that had already been covered (ie, persistence), and, once found, being able to assess the information for its relevance, helpfulness, and credibility (ie, assessability). We suggest the following design implications that support health information behavior: assessability, persistence, and searchability.

**Conclusions:**

We examined internet-based information behavior in the context of pregnancy loss, an important yet silenced reproductive health experience. Owing to the prevalence of information seeking during pregnancy, we advocate that generic pregnancy-related information spaces should address the needs related to pregnancy loss that we identified in addition to spaces dedicated to pregnancy loss. Such a shift could not only support those who use these spaces to manage pregnancies and then experience a loss but also help combat the silence and stigma associated with loss and the linear and normative narrative by which pregnancies are often represented.

## Introduction

### Background

When humans face uncertainty, disruption, stigma, or distress, helpful information encounters can be beneficial in making sense of their experiences and assist in deciding how to move forward. These encounters can occur through passive (eg, finding information without seeking information) or active information seeking. Information behavior is a concept that describes all human behavior related to information, including active seeking, browsing, and information use [1]. Social media and other internet-based tools such as search engines are important to modern information seekers, especially when information needs are not met through traditional means [2,3] and when a topic may carry a stigma in a given culture and context. This is particularly true for health information behavior.

In fact, 59% of US adults report seeking health information on the internet; 39% use the internet to determine their diagnosis, and 53% of these *internet-based diagnosers* use the information found on the internet in discussions with clinicians [4]. It is well-established that individuals experiencing health conditions (which can be disruptive, distressing, and stigmatizing) use internet-based tools to assist in managing their experiences. For example, prior work at the intersection of health and information behavior provides insights into information seeking [5], social support exchange [6,7], and how to design for better search [8-10] or educational experiences [11], including the utility of internet-based spaces in a range of reproductive health contexts (eg, infertility [12,13] or pregnancy while diabetic [14]). In this work, we examine health information behavior (including seeking, sharing, using, and the impact) as mediated and facilitated through internet-based spaces and tools in a key reproductive health context—pregnancy loss—for which such behavior remains to be explored.

Pregnancy loss is often colloquially referred to as *miscarriage.* By *pregnancy loss,* we mean the unintended loss of a desired pregnancy at any gestational stage. Pregnancy loss is an important context for investigating information behavior as it is distressing, stigmatizing, disruptive, and traumatizing for many. It challenges people’s identities as expecting parents and changes interpersonal relationships but is generally absent from societal narratives of grief or reproduction [15]. Furthermore, individuals experiencing pregnancy loss report negative interactions with medical providers, family, and friends [15]. We aligned our work with that of Layne [16], a feminist anthropologist, and Andalibi [17], a human-computer interaction (HCI) researcher, who argued that pregnancy loss should be included in feminist reproductive health discourse and pregnancy-related technology design, respectively. Indeed, the analysis of pregnancy-related mobile apps by Andalibi [17] illustrates the lack of consideration for pregnancy loss as a possible outcome of pregnancy in most apps that individuals with pregnancies use and argues that this reinforces linear, normative narratives about pregnancy and is marginalizing and harmful. The sociocultural context surrounding pregnancy loss leaves many without basic information about it, which can complicate and hinder coping.

Prior information behavior research concerning stigmatizing and distressing experiences [2,18,19] has explored the challenges people face when engaging with authority figures (eg, care providers) and how they can leverage internet-based information to fill needs not met in clinical encounters. Such work provides insights into the information that specific groups seek [20,21], how privileged and other communities differ in their use of information to inform their care [22], and the benefits and challenges individuals face when finding information on the internet [12,21,23,24]. With respect to pregnancy loss, prior work has examined topics such as social support and self-disclosure at the intersection of pregnancy loss and technology [25-27], showing how internet-based spaces are sites for support exchange, sensemaking, and validation or invalidation [28-30]. A recent study provided insights into information needs after loss, including general information about loss, counseling resources, others’ experiences, and information from providers [28]. We built on these works to explore the dimensions of information behavior after pregnancy loss in relation to existing internet-based spaces, the needs these spaces help meet, challenges they pose to individuals enduring a pregnancy loss, and outcomes.

We conducted interviews with women in the United States who had recently experienced a pregnancy loss and who used social media. We reported on 3 themes related to needed information types, information-related concerns, and information outcomes as mediated through internet-based spaces and tools. We interpreted these themes by drawing from conceptions of information use synthesized by Kari [31]: information search, knowledge construction, information production, applying information, and the effects of information. We found two main categories of information need—evidence-based and experience-based information—echoing health information needs research in other contexts [32,33]. Participants’ main information concerns were related to their ability to find information and their ability to assess the credibility of the information they found. Finally, we found that internet-based information encounters could lead to not only finding answers to questions but also learning what questions to ask and sometimes how to advocate for oneself in clinical encounters or even consider advocating for oneself as an option.

By examining information behavior in relation to experiencing personal uncertainty, disruption, stigma, or distress, we can better understand the ways people go about meeting their information needs, thus uncovering the challenges and benefits they face in day-to-day information encounters. This knowledge will (1) allow technologists and researchers to design technologies that meet people’s information needs in times of distress and fit into their existing practices with the potential to positively affect their well-being and (2) improve clinicians’ understanding of the pregnancy loss experience.

It should be noted that we advocate that pregnancy loss is not just a women’s health topic—individuals not identifying as women can also experience pregnancy and loss, and these experiences are important. In this study, although our recruitment efforts were not limited to individuals identifying as women, all participants were women. Therefore, we used this framing and terminology throughout this paper.

### Related Work

#### Information Behavior

Wilson [1] presented a general model of information behavior comprising information need, seeking, processing, and use and consideration of the role that an individual’s environment can play in their information behavior. The model by Wilson [1] has become a foundation for researchers exploring information behavior. However, Wilson [1] only accounts for when one has a known information need. Erdelez [34] extended the information behavior theories to encompass the processing of information that one was not actively seeking, termed *information encountering*. This concept allows for the exploration of the use of technologies in an information-saturated environment [34-36].

Prior scholarships have examined information behavior by examining information use. Kari [31] conducted a systematic literature review on information use within the information studies field and outlined 7 major conceptions that we draw from in interpreting our findings. In this paper, we explore the health information behavior of individuals who have experienced pregnancy loss and seek information on the internet. These conceptions, proposed by Kari [31], are relevant to our study of information behavior related to pregnancy loss:

- Information search: the processes of information seeking and information retrieval
- Knowledge construction: mental constructs are shaped or designed to function as a basis of thinking
- Information production: creating an expression of knowledge, which others can also observe
- Applying information: information functions as a resource in some processes
- Effects of information: changes brought about by information

HCI scholarship’s examination of information behavior has mainly centered on the concepts of information need [28] and information search (eg, how social contexts, environments, specific disabilities, or even mood can influence individuals’ information-seeking behavior [8-10,37,38]). In this study, we recognized the need to go beyond information seeking and consider information use concepts.

#### Health Information Behavior and Internet-Based Spaces

Health information behavior encompasses the general model created by Wilson [1] but with the information component being health information, which includes how individuals seek information about their health and health risks and examines their engagement or disengagement with health information in connection to information use concepts [31,39]. Health information seekers, both offline and on the internet, admit a heavy reliance on medical professionals for information. Medical professionals are often the first choice; however, the prevalence of internet-based resources and an optional social component has made internet-based health information seeking widespread in the United States [40,41]. Marginalized and stigmatized communities may especially benefit from finding additional health information, which they can present to their medical professionals to counteract language barriers, bias, and general dismissal [20,42]. Therefore, these groups are primed to become innovative information seekers, as they are likely to turn to and trust outside sources of health information [20-22]. Internet-based spaces can provide sources for information and support for those who are denied it or who do not sufficiently trust sources such as medical professionals to consult them [27,43,44].

Prior research has identified two types of health information: expertise-based information, which is produced by medical professionals, and experience-based information, which refers to information shared based on subjective first-hand health experiences [32]. People use both evidence-based health information and experience-based health information found on internet sites, social networking sites, and blogs [33]. Experience-based information also affects people’s health behaviors [33]. Examining health information behavior may allow us to better design health information needs.

Previous scholarship has emphasized investigating the behavior of medical professionals to better design future technology that can assist in streamlining their processes [45-48]. Noted as early as 10 years before this study [49], investigating the context of health information searching and use by individuals experiencing health conditions is also necessary to design information spaces on the internet in support of health. There have been developments in research on consumer health information behavior, even if not framed using information behavior theories, with communities such as older adults [50], pregnancy (excluding loss) [51,52], and HIV information seekers [24]. There remains a need to examine the breadth of health information topics, depth of health information seeker communities, and varied behaviors and outcomes once information is discovered. A context that is in and of itself important but that can also teach us about other stigmatizing and marginalized health experiences is pregnancy loss.

#### Pregnancy, Pregnancy Loss, and Information Behavior in Internet-Based Spaces

Information behavior scholarship related to coping with pregnancy loss is scarce, even as scholars examine the information behavior of individuals with pregnancies. Women with pregnancies use internet resources to manage pregnancies [53,54]. In fact, social media and other internet-based resources play an important role in the information needs of expectant individuals in the United States [53-56].

The information needs of individuals with pregnancies have been a research focus [57,58], although only with generic mentions of pregnancy loss, with few exceptions [28]. Similarly, design research [59,60] in the pregnancy space has largely not accounted for pregnancy loss. The significance of pregnancy loss is often minimized and rendered *invisible*. Gold et al [61] were among the first to examine internet-based support seeking of individuals who have experienced pregnancy loss, finding that internet-based support can provide a safe haven from in-person stigma and revealing a preference for moderators within these spaces. This highlights the need to understand pregnancy loss–related information needs and behavior, especially as there is no unique way in which loss is experienced [28].

We focus on pregnancy loss to examine how individuals who are enduring a loss use internet-based spaces to meet their information needs and identify the challenges and outcomes they face in this process. We effectively examine information behavior mediated through internet-based spaces for individuals enduring a pregnancy loss. Pregnancy loss, in this sense, is more broadly situated within both reproductive health and women’s health.

Pregnancy loss is common, occurring in approximately 20% of the identified pregnancies [62]. It is linked to stigma, mental health challenges, and shifts in identity and personal relationships [15]. It is also difficult to find support because of the stigma, shame, and guilt attached to it, although it tends to be traumatizing [63]. Not only is the experience absent from dominant pregnancy narratives [16], but also grieving such losses is not socially acceptable, leading to inconsiderate or unsupportive reactions from others [15]. Research at the intersection of pregnancy loss and technology has examined how and why people choose to disclose it on social media [26,27,64], how and why others respond to such content [65], the outcomes of such disclosures [25], pregnancy-related apps’ inclusion of pregnancy loss [17], validation seeking on the internet after a loss [30], and information needs [28]. Kresnye et al [28] found that forums, Facebook, and blogs are common internet-based sources after pregnancy loss and that people needed general loss-related information, counseling resources, information about others’ experiences, and information from medical providers. However, they also found challenges such as difficulty in locating resources and stigma. We have built on such work to examine what needs are met by existing internet-based spaces and what challenges they provide. We address the following research question: What are information-related processes (eg, needs, challenges, and outcomes) for individuals enduring pregnancy losses, and how are these mediated through internet-based tools?

## Methods

### Recruitment

We conducted 9 remote in-depth semistructured interviews with individuals who had experienced pregnancy loss or losses within the past 2 years (to facilitate recall and similar social media landscape). Interviews averaged 92 (range: 80-104) minutes. In this study, pregnancy loss was defined as the unintentional loss of a pregnancy at any gestational stage. Research has suggested that the gestational stage of loss is not a causal factor in the severity of grief and coping experience [66]; therefore, we did not screen participants based on the loss stage. We used a screening survey to purposefully [67] sample participants who varied in experience, demographics, and technology use to the extent possible. The screening survey was shared on the lead researcher’s social media accounts and, from there, beyond their network. This choice was informed by prior work [68-70] that used social media for reaching hard to reach populations. This was also appropriate as a criterion for inclusion was social media use. We do not know how many times the link was shared or who shared it. We did not ask participants to share the study call with those who may be eligible to participate.

### Ethics Approval

This study was approved by the University of Michigan Institutional Review Board (HUM00156077).

### Screening Survey

To qualify, respondents had to meet the following requirements: live in the United States, have experienced a pregnancy loss within the past 2 years, be a social media user, and be aged at least 18 years. Only respondents who met these criteria went on to the next stage of the screening survey, which included questions about the general use of internet-based platforms, internet-based platforms used in connection to pregnancy loss, needs after a loss to assist in processing, the month and year of the most recent pregnancy loss, age, self-description of gender, race, ethnicity, status within the LGBTQ (lesbian, gay, bisexual, transgender, and queer or questioning) community, self-description of whether they had children, relationship status, education level, household income, primary religion, and residence in an urban area (>50,000 residents) or rural areas (<50,000). We asked respondents to self-describe whether they had children or not (and how many) as this can be an emotionally charged question for those who have experienced pregnancy losses and to allow for diverse ideological views on the matter.

The screening survey received 49 responses. After survey completion, the lead researcher contacted respondents with an internet-based consent form, additional study information (eg, tools and devices they would need access to during the interview), and scheduling options. A total of 9 people completed this process and were included in the study. Participant ages ranged from 31 to 42 years. Of the 9 individuals, 8 identified as Caucasian or White and 1 as Black American; 5 identified as having children, 2 stated that they had none, and 2 chose not to answer. All identified as women, and all had some college education, with most having an advanced degree. Most were married, and one of the individuals identified as single. Of the 9 individuals, 1 identified as LGBTQ and 8 as heterosexual and cisgender. Of the 9 individuals, 1 made <US $50,000; the rest had an income of ≥US $75,000. Most lived in urban areas, and one of the individuals lived in a rural area. Table 1 presents the participants’ information.

**Table 1:** Participant information.

Characteristics	Participant ID								
	P1	P2	P3	P4	P5	P6	P7	P8	P9
Age (years)	31	34	42	32	37	30	39	31	37
Gender^a^	Female	Female	Female	Female	Female	Female	Female	Female	Female
Race^a^	Caucasian	Caucasian	White	Caucasian	Caucasian	Black	White	White	White
Ethnicity^a^	White	Adopted	Ashkenazi Jewish	Jewish	White	Black American	White	White	White
LGBTQ^b^	No	No	No	No	No	Yes	No	No	No
Children^a^	No answer	2	1	0	1	No answer	1	1	0
Relationship status	Married	Married	Married	Married	Single	Married	Married	Married	Married
Education	Graduate degree	Graduate degree	College	College	Some college	Graduate degree	Graduate degree	Graduate degree	College
Income (US $)	≥75,000	≥75,000	≥75,000	≥75,000	30,000-49,999	≥75,000	≥75,000	≥75,000	≥75,000
Religion	Christian	Catholic	Jewish	Jewish	Catholic	Atheist	Agnostic	Nothing	Atheist
Rural or urban	Urban	Urban	Urban	Rural	Urban	Urban	Urban	Urban	Urban
Technology uses	FB^c^, FSG^d^, IG^e^, and fertility tracking apps	FB, FSG, IG, and fertility or pregnancy apps	FB, FSG, IG, and TW^f^	FB, IG, Snapchat, pregnancy apps, and forums	FB, FSG, IG, TW, and fertility or pregnancy apps	FB, IG, TW, and pregnancy apps	FB, FSG, IG, TW, fertility apps, and forums	FB, IG, and infertility or pregnancy apps	FB, FSG, IG, pregnancy apps, and forums

^a^Responses to questions about gender, race, ethnicity, and having children were open ended to provide flexibility for self-descriptions. For others, we used predefined choices with an option to self-describe.

^b^LGBTQ: lesbian, gay, bisexual, transgender, and queer or questioning.

^c^FB: Facebook.

^d^FSG: Facebook support groups.

^e^IG: Instagram.

^f^TW: Twitter.

### Interview Overview

Participants chose their preferred remote tool (eg, Skype or Google Meet) as long as it allowed viewing our screen when shared (required for a portion of the study that was not used in this paper). To preserve participants’ privacy and facilitate their comfort, only audio was recorded.

### Interview Guide Overview

First, participants were asked about their life when they found out they were pregnant and what followed. Next, participants were invited to 3 illustration tasks, with their choice of words or drawing on paper, their general social media use, and their technology use during pregnancy and in relation to pregnancy loss, respectively. After each illustration, participants were asked to photograph their work and send it (via email and text) to the interviewer during the call. They were then asked to provide detailed verbal descriptions of their submissions. Visual elicitation methods often help participants articulate mental models associated with sensitive topics, facilitating flexibility and reflection [71-73]. Studies on pregnancy support networks [74] have used similar methods. We emphasized that only *they* needed to understand their submission and that we would ask them to explain it to us later.

Next, we focused on understanding participants’ needs for coping and support after loss and how these needs were or were not met, including through the use of technology. Finally, participants used paper and pens to outline an ideal support system and then shared and explained these to us, as in the aforementioned activities. Here, we focus only on the results of information behavior.

### Analysis

We recorded the audio during interviews and transcribed them for analysis. We conducted a qualitative analysis using open coding, followed by axial coding [75]. That is, we did not base our analysis on pre-existing codes as it was important to center participants’ voices and pregnancy loss.

Guided by our research question, the first author (who also conducted the interviews) developed initial codes by open coding 3 interviews. They iterated these codes and refined them using memoing in the process. Then, a team member trained in qualitative analysis was given the data and codes who met with the first author to discuss the definitions. The team member spent several weeks familiarizing themselves with all data and codes, discussing them weekly with the first author. After gaining a reasonable understanding, the team member independently applied the codes to the same 3 interviews coded by the first author. They created no new codes, despite keeping an eye out for new observations. The first author and the team member discussed any divergence in the coding of these 3 interviews to ensure mutual understanding. Then, the team member coded the rest of the data, discussing the interviews and code applications with the first author weekly. The first author then assessed the codes and excerpts to ensure consistency and grouped codes into larger categories. For example, codes for various information needs were grouped under the umbrella of information needs. The authors then refined the observed themes and discussed them in relation to the Kari [31] framework; in other words, we did not set out to use the Kari [31] framework in our study as we did not know what would be important to participants; we collected the data, analyzed them, and consulted the work by Kari [31] to interpret and organize our resulting themes. In this paper, we report themes related to our research question, focusing on information behavior.

## Results

### Overview

We report on our findings in three main themes related to the participants’ information behavior: (1) needed information type, (2) information-related concerns, and (3) information outcomes. In interpreting these themes, we draw connections to the five information use conceptions conceived by Kari [31], presented in the *Information Behavior* section: knowledge construction, information search, applying information, information production, and effects of information. We describe how these concepts illuminate participants’ use of internet-based spaces (ie, forums, pregnancy apps, search engines, and Facebook groups) for information related to pregnancy loss and coping, grieving, and sense making.

### Knowledge Construction and Needed Information Types

#### Overview

An unmet need to gain knowledge motivated participants to turn to internet-based spaces to seek information. Individuals who are likely to face discrimination or stigmatization in health care settings often turn to other sources of information [2,3]. Our findings suggest that the main types of information participants sought fell within these categories: (1) science and evidence-based information about pregnancy loss, including information about the individual with pregnancy and their partner, and (2) experience-based information, including information about pregnancy-related medical conditions and others’ experiences with pregnancy loss. According to the concept of *knowledge construction* by Kari [31]*,* individuals turn to information seeking (ie, information search) to resolve unknown or unsure knowledge and enter the process of developing new knowledge, ideas, and beliefs.

Information sources for these needs were others’ stories and experiences (experience-based) and scientifically oriented information (evidence-based), the categories in which knowledge construction [31] occurred. Notably, P9 said, “First and foremost I would say, I needed...information and...to hear other people’s stories.”

Similarly, 2 broad types of information paths were identified; P8 noted the following:

So then there’s the two paths...on the one side was that the evidence based in...things that were actually true like journal article or something that had qualitative and quantitative research...and that, in some ways, that led to this down arrow of information, which...was helpful...at times. But then after you become a statistic like losing two...sometimes the research doesn’t mean as much anymore because you realize that you can be a two percent....

P8 continued by stating the following:

But then on the other side was the real experiences and that’s what I would find through some of those forums...that in some ways it didn’t lead to that evidence-based information, but it led to in some ways validation.

Other people and academic, evidence-based information were important for P8’s experience and were complementary. In fact, many differentiated between medical, evidence-based information, and other types. For example, P7 noted a distinction between the 2 information sources:

And I think...the practical information doesn’t necessarily have to be medically informed. Having towels under you when you sleep doesn’t need to be medically informed.

This speaks to information that one would need that is not necessarily sourced from a medical professional but is still key. In what follows, we delve deeper into the types of information that participants sought on the internet.

#### Science- and Evidence-Based Information Needs

Participants often felt that medical professionals did not adequately meet their information needs:

I think, if it were to happen to me again, I would wanna talk to my OB at length, like, “Why is it that some people do it, some people don’t?”...stuff like that, being able to know specifically related to medical procedures, under what circumstances should one have a D&C? What are the implications of it? ‘Cause I’ve heard that there’s some negative side effects.

P7

P7 had questions not answered by her provider about dilation and curettage. She turned to internet-based spaces such as support groups, forums, and search engines.

Medical information needs were also informed by the gap between the participants’ needs and their level of trust in their medical care provider. Combined with the relationship aspect is a hesitancy and inability to reach out to their medical care provider when concerns may be viewed as trivial but are nonetheless crucial to participants’ experiences. Seeking evidence-based information via Google and other internet-based resources such as online support groups and forums was a way of complementing the information received from medical professionals.

P1 spoke of the role that her provider could have played to meet her information needs but did not:

I think that would have done wonders. Having my doctor able to answer all the little insane questions I would have all the time because when you’re feeling things that you’re not used to feeling...I automatically thought something was wrong...Like oh, this is happening to my body, let me Google it because I can’t just call my doctor every five minutes. So, having access to a healthcare provider that doesn’t mind you badgering them.

Obtaining knowledge about loss before both pregnancy and loss was also important. A lack of such information increased confusion and difficulty when the loss occurred. As we see in P5’s remarks, being kept in the dark by medical providers about the risks for and signs of pregnancy loss prompted her to seek information and others’ stories via Google:

When I went into the first pregnancy, I was very oblivious to a lot of stuff...I wish I would have had the knowledge, either from the group or by reading, researching, looking things up...I just assumed everything’s great, you’re pregnant...my doctor pretty much kept me in the dark about it. He would just, “Okay. Everything’s fine. Everything’s fine.” When, in reality, it wasn’t. I had to learn that the second time around from other people.

For her first pregnancy, P5 trusted that her health care provider had shared the needed knowledge and that trust contributed to P5 not looking for outside information. However, after experiencing the first pregnancy loss, P5 blamed herself for not independently seeking evidence-based information. P5 had unmet medical information needs, which were only learned after her experience with pregnancy loss. P5 expressed how much she would have benefited from the missing knowledge in coping with the first pregnancy loss. These accounts align with the finding by Kresnye et al [28] that individuals tend to attempt to find information after a loss occurs, not before it.

Some participants also noted an unmet need for information from their partners. This could take the form of resources for conveying the information they received from a medical provider to their partners and specific signs a partner could look out for.

P6 said the following:

I think, also information to help the partners. Cause like, I would go to my husband...but he didn’t have any more information than I did. And...we’re both highly educated...But...all the information we had, didn’t really prep us for...understanding miscarriage.

P2 discovered that internet-based groups could help fill that need:

It was also nice for my husband because...I don’t do a very good job of explaining my physical. I tend to write everything off. I’m like “I’m fine.”...And so it was nice for him to be able to read what I was physically going through since I was just saying I was fine. And it was helpful because then when he did read it, he would say “Stop doing the dishes. Go sit down.” “Don’t make dinner, I’m going to pick something up tonight.”

These quotes showed that P6 would appreciate guidance from medical professionals for partners of individuals navigating pregnancy loss, whereas P2 found guidance on how to express the toll of pregnancy loss to her husband and gain the support she needed from him. While some participants viewed that they experienced the loss *together* with their partner, the partner could not always be helpful partially because of lack of information. Partners can be caregivers and supporters in pregnancy journeys [76], and we suggest that future work should further consider partners experiencing losses as part of those journeys.

The turn to internet-based spaces for knowledge construction often stemmed from the unavailability of information from health care providers, whether because of participants’ hesitancy to trust health care practitioners or the health care practitioners’ unwillingness to provide it. What Kari [31] refers to as knowledge construction is the processing of information to develop new knowledge or validate unsure knowledge. Science- and evidence-based information related to pregnancy loss was an unmet need high in priority to participants, but so was practical information from others’ experiences. In the next section, we address the importance of the latter information type in internet-based spaces and the comfort offered by such knowledge.

#### Experience-Based Information Needs

A need for medically related information, which may be considered more reliable when the provider has first-hand experience or shared cultural views and identities, related to specific medical conditions or physical experiences connected to pregnancy loss. This information, sought in spaces such as blogs or internet-based support groups, was helpful, as these concerns may have been thought of as trivial by health care providers.

P3 described a need that may not easily be met by medical professionals:

But I feel like a lot of my issues...are kind of cultural...and that being able to speak about them in a group that’s more culturally similar...like Jewish rituals, superstitions...being able to have other people’s input and guidance and what worked for them would have been nice.

P3 wanted to find information sources who understood her cultural beliefs and had insight into dealing with pregnancy loss as a Jewish person. Determining whether a medical professional could provide this insight may not be possible, so she turned to digital spaces.

Similarly, P6 emphasized the importance of receiving information from individuals who share similarities, particularly when one is a part of a group that can often receive less than the standard of care from medical professionals.

Well...there’s been a series of studies...about how, oh black women are nearly four times more likely to die giving birth...more likely to be denied pain medication...their babies are more likely to die before the age of one. So that’s something I think about as a researcher. Like the gap between logistics, because all of those are just the outcomes of logistic models...you have to understand that statistics are not a good way to tell the story. So, I started just paying attention more to women, black women telling their stories. And...anecdotes aren’t data. But those stories do matter....

P6

P3 and P6 turned to internet-based spaces in search of information that they deemed critical to their specific experiences of pregnancy loss based on culture, race, medical beliefs, and medical statistics. Individuals who have experienced pregnancy loss may not have access to medical professionals who can provide information that speak specifically to such cultural or racial issues.

Often, medical information could originate from social ties with relevant personal experiences (as described above) or professional expertise. For example, P2 mentioned needing dietary information after her loss, which a family member who had a relevant scientific background was able to provide:

My kid’s godfather...studies gut bacteria and he was like “Well here are some things that can actually help with...absorption of nutrients” And so that was really helpful. If I’m taking all these supplements, then I want to know how I’m actually absorbing them.

These examples illustrate participants’ needs to find information not only about pregnancy loss broadly but also about the variety of relevant conditions that may not be shared across all individuals experiencing pregnancy losses.

Participants also reflected on the need to know the basics of pregnancy loss symptoms, what to physically expect, and how to practically prepare:

I wish a girlfriend would have been like, hey, you should lay down a bunch of towels in the bed because the second day is worse...it would have been very useful...But people don’t tell you this...Little tiny practical tips...Nobody would hurt from...sleeping on towels.

P7

In addition to information aligned with one’s experience, condition, and identity and culture, participants noted that information from others who had experienced losses could also be practical.

Another example included not only looking for symptoms of loss but also gaining basic information on the stages of pregnancy:

Basically Glow was not only a way for you to track your symptoms of your cycle...Do you have your cervical discharge?, and any kind of other symptoms that you have and if you’ve gotten a positive OPK. The forums are also helpful because I didn’t know a lot at the time. I didn’t know that it takes 6-12 days for an egg to implant and 2-3 days...to get a positive after that.

P4

This basic information on the symptoms of loss and pregnancy stages may be overlooked by medical professionals as obvious or trivial, which makes internet-based groups an avenue for education. Furthermore, we see that some information needs after a loss tend to be about future pregnancies, which are necessarily positioned within one’s pregnancy history, including the loss.

A combination of evidence-based, identity and culture–aligned, and practical information from others who have experience with pregnancy loss made up most of the information that participants sought or needed. However, participants also noted finding information that they would have preferred from their evidence-based sources that were not readily available. For example, some were able to find information on forums that were not available through Google searches (ie, deleted by clinic businesses) and may not be readily shared by health care professionals. For instance, P7 shared the following:

Those forums were super-duper useful. And...that information wouldn’t be out there at Google, because these fertility clinics...don’t want people to actually know how much these things cost. And...They clean the web of reviews and stuff, because it’s a business.

In this section, we described the information needs that health care providers were either unwilling or unable to meet and the sources that participants, in turn, used to construct knowledge. Our analysis highlights two main overarching types of information that participants sought: (1) science- and evidence-based information about pregnancy loss, including information about the pregnant individual’s partner, and (2) experience-based information, including information about pregnancy-related medical conditions and others’ experiences with pregnancy loss, especially those aligned with one’s culture and identity. Participants were able to address many of these information needs in internet-based spaces but would appreciate and benefit from health care providers’ input regarding the credibility of medically related information and their assistance in conveying their knowledge to partners. The next section covers specific concerns that participants expressed related to pregnancy loss–related information encounters in internet-based spaces.

### Information-Related Concerns in Information Search

#### Overview

Our findings suggested two main concerns in information encounters: (1) being able to find information and (2) determining the credibility of information. These are aligned with the *information search* concept of Kari [31], as the ability to use information or information resources is directly connected to the feasibility of searching for and accessing information, which makes these concerns important for designing internet-based spaces. Persistence (ie, “the extent to which a platform affords the continued availability of content over time” [77]) and searchability (ie, the extent to which a platform affords users the ability to search for content using search terms) together afforded timely access to information.

#### Challenge 1: Ability to Find Information Shaped by Persistence and Searchability

Persistence and searchability often went hand in hand. Easy access to and almost real-time return on information was important. Posting a question on an online forum or support group and waiting for responses was not an appropriate method to meet some needs.

For example, P7 used search engines to quickly find information from old forum posts:

For me at that stage, googling for info went infinitely to the top...I didn’t necessarily wanna talk to a human about what I was experiencing...I didn’t wanna wait for a human to respond. I wanted in that minute to be like, “How much blood loss is too much? When should I call my doctor?” I need this immediately.

Participants appreciated the ability to search for information on the internet, find it quickly, and be able to access it without posting about their own experiences, which can be difficult during moments of distress. The benefit of finding and reading available posts and answers rather than posting oneself is seen in P1’s response:

I had lots of questions. The cool thing about the Facebook group is not only do you have everything that people are posting right now, but you can go to the top and you can type in a specific thing. And then all the posts that have to do with that come up.

P7 described the importance of the interfaces in supporting search, evidence-based information, and archived information:

Medically informed information with citations...in terms of the actual interface it has to be super-duper searchable, with a very good search. That’s one of the downsides to app-based stuff is that you can’t go into the Ovia pregnancy app for example, and search 12 weeks or something...Also...making sure that it’s archived, so that...I can see here’s something someone posted three years ago that was similar to my situation, so I don’t have to wait for answers in the moment...there was quite a while that the searchability of Facebook was really poor, especially on mobile...that’s really unfortunate.

Participants found value in archived information, collective experiences, and searchability. Being able to search for relevant information required an effective search functionality and permanent storage that allowed participants to draw from the vast amount of experience-based and evidence-based information shared over time. Internet-based spaces can provide swift access, in contrast to contacting, scheduling an appointment, and then conversing with health care providers. If an internet-based space maintains truly usable search functionality and stores information in a persistent manner, it can be a resource for information needed at particular moments. As seen in P7’s comment on the difficulties of searching in apps, when persistence and searchability are not maintained, information seekers turn to other spaces to meet their information needs.

#### Challenge 2: Determining Information Credibility

Facing misinformation or information perceived as less credible or difficult to assess was another challenge. Participants often turned to internet-based forums for information about specific conditions related to loss. There, they encountered the challenge of assessing what information was credible and what was not.

P1 described this problem as follows:

As far as Baby Center, I used that a ton right after the ectopic pregnancy because I didn’t know who else to ask...I found a lot of helpful things...but...you also find things like things that don’t seem to be helpful...oh, I heard my neighbor’s friend’s sister had this response. Like well, is it that really...fact?

As another example, P4 said, “[That was] really helpful in getting educated, but there’s also a lot of garbage in there, which I found out once I moved over to the Ava group.” Although P4 was able to gain some useful information in a Facebook group, she also found misinformation, which became apparent to her once she moved to a space that she thought included less misinformation.

The amount of misinformation led some to leave internet-based forums altogether*:* “I did look at the forum but they drove me nuts and I stopped looking at them. They’re a lot of misinformation*...*” [P6]. This is problematic as people come to these spaces to find information and social support; leaving means that they will not be able to access other types of support either. Ideally, people who join these spaces would stay for some time, both to find support and information for themselves and contribute to support others.

Participants found it difficult to find medically approved information about pregnancy loss in general pregnancy forums. This was particularly challenging and a cause for confusion because of advice that was contested. To this point, P7 said the following:

But medically informed information that...I could trust...is hard to find in general forums. Those infertility forums are pretty exceptional in how knowledgeable those people are about medical stuff, but in general pregnancy forums, people are dumb as rocks.

When participants sought information on Google or forums, they noticed a prevalence of what they considered nonscientific information. P3 reflected on a way of assessing the credibility of evidence-based information shared in digital spaces:

I could imagine, in a way, an information center, where it’s not so much about feelings but the questions are more fact-based and the discussion is more fact-based. I guess you can’t really have medical advice because it’s always lawsuits. But where people are encouraged to give answers that are fact-based, like studies and what they read.

Finding credible information that one can trust could have helped P7 and P3 make sense of their experiences, plan for the next steps, and regain control that was lost through the loss experience.

A main motivator for turning to internet-based spaces was a need to gain knowledge quickly; however, that benefit can be negated when one is unable to determine the credibility of information, when the information is deemed to be not credible, or when digital spaces do not afford effective searching. In the next section, we address the outcomes of looking for and finding information on pregnancy loss in internet-based spaces.

### Information Outcomes: Applying Information, Information Production, and Effects of Information

#### Overview

Accessing information in internet-based spaces has implications for participants’ future behaviors. We found that after new knowledge construction, participants (1) used information to advocate for themselves with medical professionals and (2) had their concerns and ideas validated. These outcomes align with the following information use concepts identified by Kari [31]: applying information, which occurs when one uses new knowledge, such as raising concerns to a medical professional; information production, which involves using learned knowledge to produce information for others, such as writing a post on an internet-based space; and the effects of information, where the information influences one’s future choices. In these internet-based information spaces, participants developed tools for clinical encounters, such as learning what to ask and how to advocate for themselves. Newly obtained knowledge can also provide insights into options that are not presented or even discouraged by health care providers, in turn influencing one’s decisions.

#### Applying Information: Learning What to Ask

For some, internet-based spaces were places where participants learned what questions to ask. P9 said the following:

It’s only with finding an online community that I also found the language I needed to ask, should we be doing the following things? Should we be considering this kind of doctor? This kind of testing?

This is important, as the stigma surrounding loss contributes to less discourse and education about the topic [26,63].

Learning without asking was also a benefit, described by P5 as follows:

At the beginning of my pregnancy, there was a lot of warning signs that I wasn’t aware of: the heartbeat was low, I had low progesterone. There’s so many different things that I didn’t pick up on, because I just wasn’t aware. And being part of this group...helped me become aware, because these women had similar stories.

This is noteworthy, as lurking is often associated with negative connotations [78]; however, we suggest that it can serve as an effective learning strategy.

Overall, internet-based spaces and tools not only provided answers but also inspired participants with questions they could ask health care providers. Knowing what to ask and what to look for can make an individual more informed and lead to changed behavior. Participants expressed that they had used or would use knowledge found in digital spaces to obtain the needed information in conversations with medical professionals and prepare an outline of warning signs to try to avoid a future pregnancy loss; these examples align with the Kari [31] concept of applying information.

#### Information Production and Effects of Information: Changed Behavior

Internet-based spaces also become spaces for sharing one’s own experience to inform others. P3 used an internet-based space to share information that she thought was needed and unavailable from other sources:

The other thing I’ll post about is with my third miscarriage, I had the choice of getting...a different procedure...that most women...don’t realize is an option...So that’s kind of my soapbox...I’ll often post about it...if I feel like I can offer relevant, helpful information.

In this case, P3 produced information to help others advocate for themselves by contributing experience-based information that she knew would otherwise be unavailable to others. This is an example of the information production concept of information use by Kari [31].

Another example is found in P2’s choice to remain in an infertility group as a source of information for others:

I debated...whether or not to stay, but I feel like if anyone was going through what I went through, it took so long for my doctors to diagnose the problem that...I’d want to help somebody, because it was a simple blood test that nobody bothered to run for a year.

P2’s role in the infertility group changed from information seeker to information producer, a source of experience-based information.

As noted in earlier sections, internet-based spaces such as Facebook groups or forums fill an informational gap that can occur when health care professionals are not available or refuse to meet information needs and when other internet-based resources (eg, Google) are unavailable or inaccessible (eg, information related to fertility clinics deleted from Google by clinic businesses). Here, we see how the knowledge gained in these spaces can lead to more informed decisions. As an example, P9 shared a story about finding instructions about in vitro fertilization injections, which led to behavior change; this content comprised questions that she did not get answers to from her provider but instead from other patients, including how to advocate for herself with her provider:

But in that group...especially once we started IVF...people share tips about how to make injections less painful, how to get the right angle...I know it can sound...ill-advised to get medical advice from people who aren’t doctors, but a lot of the advice is more about how to advocate for yourself as a patient...something that I have learned almost exclusively through being in these groups.

P5 offered another example of learning to advocate for oneself:

I had to be put on progesterone and I had to advocate for myself with my doctor...to put me on whatever progesterone they could...to maintain this pregnancy, which I carried to full term...That was, again, something I learned from the women in those groups...You definitely want to be your own advocate, and self-advocate for yourself...

The above examples align with the concept of *effects of information* by Kari [31], where the information participants found in these internet-based spaces influenced their future choices, including how to navigate advocacy with a physician.

## Discussion

### Principal Findings

Our findings highlighted the following three novel themes associated with information behavior after pregnancy loss as mediated through internet-based spaces: (1) needed information type, (2) information-related concerns, and (3) information outcomes. These findings are significant as a first step in designing internet-based spaces to account for pregnancy loss information behavior. We found that participants shrank the knowledge gap regarding what to ask by learning what information others found worth knowing. They bridged language barriers by using information from internet-based spaces to supplement suggestions from medical providers, and the barriers of social stigma, cultural taboo, and lack of social and economic capital were to some extent offset by participants’ ability to advocate for themselves, stemming from the confidence gained from interactions in internet-based spaces.

We showed how internet-based information spaces help alleviate some information gaps for individuals experiencing pregnancy losses and supplement information gained in clinical encounters. Nevertheless, digital health resources pose their own obstacles: people need to be able to find the information, understand and determine its credibility, and know how to use it. In addition, primary barriers to health care for women from stigmatized groups include knowledge gaps such as not knowing where to find information and what resources are available or what information to ask for, and a lack of understanding between patients and providers [79], which we also saw examples of in the case of pregnancy loss.

### Information Behavior, Health, Pregnancy, and Pregnancy Loss on the Internet

In our dedication to fully engage with information theories beyond search and need, we outlined our analysis in connection with the information use concepts by Kari [31]; however, our participants’ responses also related to other information models. The Wilson [1] model remains relevant, as seen in participant responses first in their expression of unmet information needs (ie, information types), which led to information seeking on the internet, and in their insight into how the environment in which they sought information can influence their behavior (ie, information-related concerns), and finally in how they used information (ie, information outcomes). However, participants often learned what questions they *should* ask related to pregnancy loss in these internet-based spaces, which is a form of information encountering [34]. The internet, as an information-rich environment, is a source for both active information seeking and information encountering, which can occur through browsing or as a part of active seeking.

Pregnancy loss–related information seekers were similar to other health information seekers [40,41] in sharing high regard for information from medical professionals but needing additional information as well. Medical professionals are unable to meet certain personal needs that only knowledge gained from lived experiences can provide [80,81]. The main types of health information sought in prior literature in other health contexts [32,33] align with our participants’ expressed need for evidence- and experience-based information. Participants did not expect peers to act as pseudo–medical professionals providing medical expertise or interpreting medical advice but instead to offer suggestions on topics such as how to improve communication with clinicians or insight into experiences with a recommended treatment or procedure [80,81]. We note that in contrast to the documented lack of trust between individuals who are socially marginalized or stigmatized and health care providers [12,14,15], participants expressed trust in medical professionals; their concerns centered on not receiving enough information on this particular topic and possibly not knowing how to broach it with medical professionals. Nevertheless, this expression of trust in medical professionals could be because of our sample limitations as most participants were White and of higher socioeconomic status.

Even so, we extend the scholarship on pregnancy-related information behavior. Participants expressed a desire for information on pregnancy loss *early on* in the pregnancy to prepare for the possibility of complications arising, which resonates with earlier research demonstrating that individuals with pregnancies begin their search for information at the outset of the process [53]; however, research on information needs after pregnancy loss suggests that most seek this information after the loss, not before it [28]. Similar to prior research on the information-seeking (not accounting for loss) [53,82] of individuals with pregnancies, participants in our study showed a propensity for changing behavior in response to the information they discovered on internet-based spaces. Participants also demonstrated a tendency to use this information to advocate for themselves in clinical encounters, which has not been heavily reported on in expectant parents whose pregnancies do not lead to a loss [54].

Some barriers for participants were similar to those faced by other pregnancy-related information seekers: not having instruction on or assistance in conveying information to a partner and lacking targeted information for individual experiences [76]. Gold et al [61] were one of the first to examine support seeking for pregnancy loss on the internet; our findings reiterate the important role that internet-based support and resources can play. Participants in the study by Gold et al [61] also expressed a need for additional evidence-based information, feeling validated in internet-based spaces, appreciating the ready information access that internet-based spaces can provide, and the value of seeing others move on after pregnancy loss or related medical issues. However, Gold et al [61] focused on the benefits of these internet-based spaces and did not address the barriers to acquiring pregnancy loss–related information or their associated outcomes. Our findings on others’ experiences and medical providers as sources of information also resonate with the study by Kresnye et al [28]. Although Kresnye et al [28] explored challenges in accessing information, such as locating resources and self-blame, we identified other challenges with respect to accessing, understanding, and applying information related to the technical features required for effective search and assessing information credibility.

We discuss internet-based health literacy concerns and considerations for design in the following section. Pregnancy-related apps generally do not account for pregnancy loss [17], including information behavior–related needs. We suggest that future pregnancy-related and pregnancy loss–related information spaces should address the needs and challenges that we identify herein [28].

### Designing for Assessability, Searchability, and Persistence

#### Overview

An important aspect of the internet-based health literacy process is assessing data quality within an information environment to determine its usefulness [83,84]. Our participants described internet-based health literacy and data quality as concerns in connection to credibility challenges when seeking information.

The themes we identified resonated with the need to design for populations based on their specific information needs and barriers to meeting those needs, as emphasized in current scholarship on health information behavior in internet-based spaces. Individuals who have experienced pregnancy loss have information needs that, although specific to them, also share the concerns over determining credibility with others [21,85]. Our themes also resonated with current HCI literature on the needs of patients managing chronic disease in internet-based health and support groups [80,81], including consideration of the user as a whole, personal and clinical information, clear organization and the ability to search archived information, and safeguards against misinformation. The remainder of this section addresses these concerns.

#### Designing for Assessability

Our findings speak to the need to extend the *assessable design* framework [86] to the internet-based health context. Designing for assessability was first introduced by Forte et al [86], fusing information literacy concepts in various disciplines toward developing a framework for the *assessable design* of participatory information sources (eg, Wikipedia, forums, and support groups). The authors established two concepts critical for assessability by reviewing the information credibility literature: (1) information provenance, concerned with determining where information comes from, and (2) information stewardship, which refers to how an information space is maintained [86]. Assessable designs should facilitate the understanding of how the information in internet-based participatory environments is produced, how an environment is sustained, and how to contribute to the space [86]. In fact, prior research investigating the processes individuals use to determine credibility found that users are likely to refer to the source; sources are deemed more credible if the poster is a professional expert or an expert according to community status or past engagement [87-89].

Applying the assessable design framework to our results, internet-based spaces could provide users with affordances that allow them to identify and assess information sources and contributors and their credibility; for example, internet-based groups may consider adding identity tags to content producers that would enable others to assess their contributions. These recommendations resonate with prior suggestions for formal patient–provider internet-based spaces for patients with chronic diseases [80,81]; our findings extend these design needs into informal digital spaces and beyond chronic disease management to individuals managing experiences such as pregnancy loss that can be acute or chronic.

Participants expressed the need for information in two categories: others’ stories and experiences (experience-based) and scientifically oriented information (evidence-based). Individuals want to know that their information source is a knowledgeable expert, whether because of training or lived experience. Therefore, designing for the clear identification of the source can assist users in determining the credibility and usefulness of the information, as people are more likely to adjust their attitudes or behaviors in response to a message from someone deemed an expert [89]. These design directions could assist in users’ effectiveness and confidence in doing what we found they already try to do: advocate for themselves with medical professionals.

Although identity attributes should be developed together with relevant stakeholders in future work, examples could include determining whether a person has relevant professional expertise. Encouraging or enforcing the citation of sources for the information shared in internet-based spaces as part of the community guidelines is another consideration; individuals could be prompted to add references to the content they share so that others can assess those sources and the information’s credibility.

Similar to Forte et al [86], internet-based spaces could also provide users with aggregate information (eg, through simple visualizations) that show what sources contributors draw from. For example, one may go to a Facebook pregnancy loss group and see that 70% of posts link to outside sources, and out of those, 30% are academic peer-reviewed articles about pregnancy loss. This would likely provide the user with an assessment of the group as a whole. We advocate for including assessability in design considerations.

#### Designing for Searchability and Persistence

Our analysis led to identifying searchability and persistence as desired affordances for participants. For example, some participants shared successful experiences with a Facebook group’s search feature and less successful experiences with some pregnancy-related apps and forums. The point here is not to compare platforms (which requires other methods and is an area for future work) but to learn from participants’ experiences. Our findings show that people experiencing pregnancy loss need both evidence-based and experience-based information. There is a wide range of adjacent medical conditions (eg, polycystic ovary syndrome) that shape people’s loss experiences; finding information about those conditions was also important to participants. Taken together, future designs could experiment with features that allow people to tag the content they contribute as *evidence-based* or *experience-based* or with adjacent health condition labels to streamline searching for others. Although this needs further exploration, this design application would likely be well-suited for an integrated patient–provider application, as described in the study by Huh et al [81], where peers can share and discuss content with one another and consult a medical professional as needed. A possible added benefit of such a design could be the ability for the expert medical professional to share insights without compromising peer-to-peer sharing, which was an issue observed by Huh et al [81].

Such an approach could also be used to provide aggregate information about what experiences and information types are represented within an internet-based space (eg, 79% experience-based). This design approach could be combined with the approach suggested by Huh et al [81], which calls for designing systems that can suggest prior threads relevant to a user’s post, or with the suggestion of Hartzler et al [80] for profile features that detail topics a user typically discusses or posts information about.

In summary, our findings show that when there is a dire need for information, access to relevant experiences and information and the ability to assess the credibility of such information are key. The first can be supported by designing for easy retrieval and organized storage of older content through searchability and persistence. The second could be supported by an assessable design. That said, privacy considerations are crucial; for example, if contributors want their data to expire after a certain point, they should be able to easily achieve that goal. Privacy concerns are especially important when designing for information retrieval in the context of well-being in internet-based communities, which are not regulated at the same level as formal, traditional health information (eg, electronic health records). Altogether, we argue that designing for assessability, persistence, and searchability is needed to achieve dimensions of health literacy (ie, access, understand, appraise, and apply) [84]. Without these aspects of design, individuals are less equipped to find information, make informed decisions about how the information could assist in maintaining their health, and effectively communicate this information to others. Researchers and technologists could consider these recommendations when designing pregnancy-related internet-based spaces, which tend to grossly neglect pregnancy loss [17], as well as spaces dedicated to pregnancy loss. Accounting for pregnancy loss in designing internet-based information spaces will help counter the stigma many endure and make space for diverse pregnancy narratives.

### Limitations

The larger context within which survivors experience pregnancy loss shapes their experiences. Therefore, we focused primarily on the United States to examine pregnancy loss experiences in this study. We encourage researchers to explore similar topics in other countries. We hoped to include a wide range of experiences (eg, technology use and pregnancy history) and demographics such as age, race, and income level to the extent possible. We were not entirely successful in achieving diversity on all these levels. Although our sample represents a range of technology experience and pregnancy history, it was primarily White, cisgender, heterosexual, married, educated, and urban and had an income >US $75,000. Our sample’s demographics are a limitation; however, the findings advance our knowledge about information behavior after pregnancy loss. In the future, we hope to build on this work by reaching more diverse populations, for example, by partnering with community organizations serving marginalized groups and including partners or caretakers. Although an accepted recruitment practice for engaging hard to reach populations in research [68-70], the social media recruitment strategy has its limitations (eg, not everyone who would have been eligible saw our study call).

Our findings are not generalizable; however, they still hold value and contribute to our knowledge about social technologies’ roles in information behavior after pregnancy loss. In addition, rather than achieving validity through quantity, in-depth long interview studies with small sample sizes support interpretive claims achieved through the careful selection of participants who share experiences related to research questions [90]. As such, our findings are not intended to be generalizable; rather, they are presented as generative points to provide a conceptual vocabulary for describing information behavior processes. Future work may use representative samples and surveys to assess the prevalence of the identified themes in this paper.

## Abbreviations

HCI: human-computer interaction
LGBTQ: lesbian, gay, bisexual, transgender, and queer or questioning
